# Systemic Corticosteroids and the Risk of Venous Thromboembolism in Patients with Severe COPD: A Nationwide Study of 30,473 Outpatients

**DOI:** 10.3390/biomedicines9080874

**Published:** 2021-07-23

**Authors:** Ema Rastoder, Pradeesh Sivapalan, Josefin Eklöf, Mohamad Isam Saeed, Alexander Svorre Jordan, Howraman Meteran, Louise Tønnesen, Tor Biering-Sørensen, Anders Løkke, Niels Seersholm, Thyge Lynghøj Nielsen, Jørn Carlsen, Julie Janner, Nina Godtfredsen, Uffe Bodtger, Christian B. Laursen, Ole Hilberg, Filip K. Knop, Helene Priemé, Truls Sylvan Ingebrigtsen, Vibeke Gottlieb, Jon Torgny Wilcke, Jens Ulrik Stæhr Jensen

**Affiliations:** 1 Section of Respiratory Medicine, Herlev-Gentofte Hospital, 2900 Hellerup, Denmark; pradeesh.sivapalan.02@regionh.dk (P.S.); josefin.viktoria.ekloef.01@regionh.dk (J.E.); mohamad.isam.saeed.02@regionh.dk (M.I.S.); alexander.svorre.jordan@regionh.dk (A.S.J.); howraman.meteran.01@regionh.dk (H.M.); louise.toennesen@gmail.com (L.T.); seersholm@dadlnet.dk (N.S.); vibeke.gottlieb.03@regionh.dk (V.G.); Jon.Torgny.Wilcke@regionh.dk (J.T.W.); jens.ulrik.jensen@regionh.dk (J.U.S.J.); 2 Section of Cardiovascular Medicine, Herlev-Gentofte Hospital, 2900 Hellerup, Denmark; Tor.Biering-Soerensen@regionh.dk; 3 Department of Medicine, Hospital Lillebælt, 7100 Vejle, Denmark; aloekke@gmail.com (A.L.); Ole.Hilberg@rsyd.dk (O.H.); 4 Department of Regional Health Research, University of Southern Denmark, 5000 Odense, Denmark; 5 Section of Respiratory Medicine and Infectious Disease, Nordsjællands Hospital, 3400 Hillerød, Denmark; Thyge.Lynghoej.Nielsen@regionh.dk; 6 Department of Cardiology, Rigshospitalet, Copenhagen University Hospital, Department of Clinical Medicine, Faculty of Health and Medical Sciences, University of Copenhagen, 2100 Copenhagen, Denmark; Joern.Carlsen@regionh.dk; 7 Section of Respiratory Medicine, Amager and Hvidovre Hospital, 2650 Hvidovre, Denmark; julie.janner@regionh.dk (J.J.); Nina.Skavlan.Godtfredsen@regionh.dk (N.G.); 8 Section of Respiratory Medicine, Næstved Hospital, 4700 Næstved, Denmark; ubt@regionsjaelland.dk; 9 Department of Respiratory Medicine, Odense University Hospital, Odense Respiratory Research Unit (ODIN), Department of Clinical Research, University of South Denmark, 5000 Odense, Denmark; Christian.B.Laursen@rsyd.dk; 10 Center for Clinical Metabolic Research, Gentofte Hospital, University of Copenhagen, 2900 Hellerup, Denmark; filip.krag.knop.01@regionh.dk; 11 Steno Diabetes Center Copenhagen, Gentofte Hospital, 2900 Hellerup, Denmark; 12 Department of Clinical Medicine, Faculty of Health and Medical Sciences, University of Copenhagen, 2200 Copenhagen, Denmark; 13 Section of Respiratory Medicine, Herlev-Gentofte Hospital, 2730 Herlev, Denmark; Helene.Prieme@regionh.dk; 14 Department of Respiratory Medicine, Roskilde Hospital, 4000 Roskilde, Denmark; truls.sylvan.ingebrigtsen@regionh.dk

**Keywords:** COPD, exacerbation, venous thromboembolism, pulmonary embolism, corticosteroids

## Abstract

Due to frequent exacerbations, many patients with chronic obstructive pulmonary disease (COPD) are exposed to oral corticosteroids (OCS), which may be thrombogenic. We evaluated the risk of hospitalisation with venous thromboembolism (VTE) and death in patients with acute exacerbation of COPD (AECOPD) treated with long and short OCS regimens. In this nationwide cohort study of 30,473 COPD outpatients treated for AECOPD, we compared the risk of VTE hospitalisation and all-cause mortality within 6 months in OCS dose of >250 mg vs. ≤250 mg. A multivariable Cox proportional hazard regression was used to estimate the risk. The incidence of VTE hospitalisations was 0.23%. A long OCS treatment course was associated with an increased risk of VTE compared to a short course (hazard ratio (HR) 1.69, [95% confidence interval (CI) 1.05 to 2.72], *p* < 0.031). A higher risk of all-cause mortality was seen in the group of COPD patients treated with a long OCS course (HR 1.71, [95% CI 1.63 to 1.79], *p* < 0.0001). The risk of reported VTE hospitalisation was higher among AECOPD patients treated with long courses of OCS, but the absolute risk was low, suggesting under-reporting of the condition.

## 1. Introduction

Chronic obstructive pulmonary disease (COPD) is characterised by an obstructed airflow limitation that is not fully reversible and an abnormal inflammatory response of the lungs to noxious particles and gases. According to the current guidelines, the inhalation of short-acting beta-adrenergic agonists and anticholinergic agents, systemic corticosteroids (OCS) and oxygen therapy is considered the standard therapy for acute exacerbation of COPD (AECOPD) [1,2]. The long-term use of OCS is generally avoided due to the risk of adverse effects [3,4,5]. Venous thromboembolism (VTE) is a known but rare side effect to corticosteroid treatment. Several studies have investigated the association between OCS and VTE—however, not in COPD patients [4,6,7,8,9,10,11]. AECOPD patients are at a high risk of VTE during hospitalisation [12,13]. In line with this notion, an autopsy study in patients with AECOPD reported 21% of sudden deaths are caused by VTE [14]. However, it is unknown if OCS treatment is associated with an increased risk of VTE hospitalisations in severely ill COPD patients frequently treated with OCS.

Using a large and well-characterised population of severely ill outpatients with COPD, we investigated whether OCS duration in AECOPD is associated with an increased occurrence of VTE hospitalisations and all-cause mortality.

## 2. Materials and Methods

### 2.1. Study Design

This was a nationwide observational cohort study where the patients with severe or very severe COPD were followed for 6 months. The index date was defined as the date of the first OCS prescription. The patients were followed from the index date to the first occurrence of VTE hospitalisation, death or 6 months from the index date. A short follow-up time was chosen, since earlier data suggested that the risk of VTE was highest in the first month after OCS use [15]. Patients without an OCS prescription, patients with primary thrombophilia and asthma patients were excluded.

### 2.2. Study Source

The following nationwide registers were used in this study:

The Danish Register of Chronic Obstructive Pulmonary Disease (DrCOPD) is a nationwide database that contains information on the quality of treatment of specialist- and spirometry-verified patients with COPD in Denmark [16]. Since 2008, all Danish hospitals treating COPD patients have reported to the register, and the patients were assessed by a respiratory physician who confirmed the COPD diagnosis. Most of the patients from the DrCOPD registry have either frequent exacerbations and/or are known to have severe COPD. The obtained information included age, forced expiratory volume over 1 s (FEV_1_%), body mass index (BMI), level of dyspnoea (using the Medical Research Council Dyspnoea Scale) and smoking status (smoker vs. non-smoker).

The Danish National Patient Registry (DNPR) contains information on all admissions since 1977 and hospital outpatient clinic visits since 1995. Physicians coded each hospital visit with one primary diagnosis and one or more secondary diagnoses, according to the International Classification of Diseases 10th revision (ICD10) from 1994 [17].

The Danish National Health Service Prescription Database (DNHSPD) holds information on all prescriptions that have been dispensed in Danish pharmacies since 2004, coded according to the Anatomical Therapeutic Chemical (ATC) classification system. The date of dispensation, the quantity dispensed, and the formulation and strength of the prescription are all included. Pharmacies are required by Danish legislation to provide information that ensures complete and accurate registration. The DNHSPD was used for information on OCS [18].

### 2.3. Study Participants

Danish residents registered with a COPD diagnosis between 1 January 2010 and 28 February 2018 were identified in DrCOPD. The base cohort was formed by linking these individuals with prednisolone prescriptions (ATC code H02AB06) for the treatment of AECOPD in the outpatient clinic. All prescriptions were registered after the patients’ registry in DrCOPD. Patients treated with prednisone (ATC code H02AB07) were excluded, as the medication was given for other reasons. Patients were stratified into two groups: (i) prednisolone prescriptions below or equal to 250 mg (i.e., a prescription of the maximum of 10 tablets of 25 mg each), corresponding to a short OCS treatment course, and (ii) prednisolone prescriptions above 250 mg (i.e., a prescription of more than 10 tablets of 25 mg each), corresponding to a long OCS treatment course. Due to a guideline change in 2014, the recommended duration of OCS treatment for AECOPD was reduced from 10 to 5 days (37.5 mg daily) [2,3]. This definition was used in a previous publication [3]. The change of the OCS duration recommendation in 2014 made it possible to compare long and short OCS treatment courses. Furthermore, both treatments were given during their respective periods as first-line treatments for AECOPD, enabling a comparison that mitigates the possibility of confounding by indication.

### 2.4. Variables

The date of the first OCS prescription was defined as the index date. This study’s primary outcome was VTE hospitalisation (defined as deep venous thrombosis (DVT) and/or pulmonary embolism (PE)) within 6 months from the index date. The secondary outcome was all-cause mortality within 6 months from the index date. The exposure was the OCS prescription. Cancer and anticoagulation treatments were examined as effect modifiers. Hospitalisations with DVT or PE were identified by DNPR (ICD codes (I26.0, I26.9 and I80.1–I80.5 and I80.8–I80.9 and I82.2–I82.9)). The date of death was specified in the DrCOPD.

### 2.5. Statistical Analysis

For descriptive statistics, continuous variables were reported using the median and interquartile range (IQR) and categorical variables as the frequencies and proportions. In addition, the Mann–Whitney *U* test was used to compare continuous variables and the χ^2^ test for dichotomous variables.

Cox-regression was used to assess the risk of VTE hospitalisation or all-cause mortality during the 6 months the COPD patients were followed. The results were presented as the hazard ratio (HR) with a 95% confidence interval (CI). Model control investigating the proportional hazards assumption and test for linearity was performed to validate a Cox proportional hazards regression. A competing risk Cox proportional hazards model was used to address the major competing risk in this population, namely death from all causes. All analyses were adjusted for the following known confounders: sex, age, inflammatory bowel disease, cancer, former events with VTE hospitalisation, recent surgery and/or trauma, AECOPD hospitalisation 1 year prior to the first OCS prescription, smoking status, FEV_1_%, time period for prescription distribution (prior/post guideline shift), anticoagulation treatment 1 year prior to the first OCS prescription and number of OCS prescriptions. Furthermore, the sensitivity analysis investigated the modification effects between OCS use and cancer and the risk of VTE, and the modification effects between OCS use and anticoagulation treatment and the risk of VTE. Statistical analyses were carried out in SAS Enterprise Guide 9.4 (SAS Institute, Cary, NC, USA).

## 3. Results

### 3.1. Descriptive Analyses

**Figure 1:**
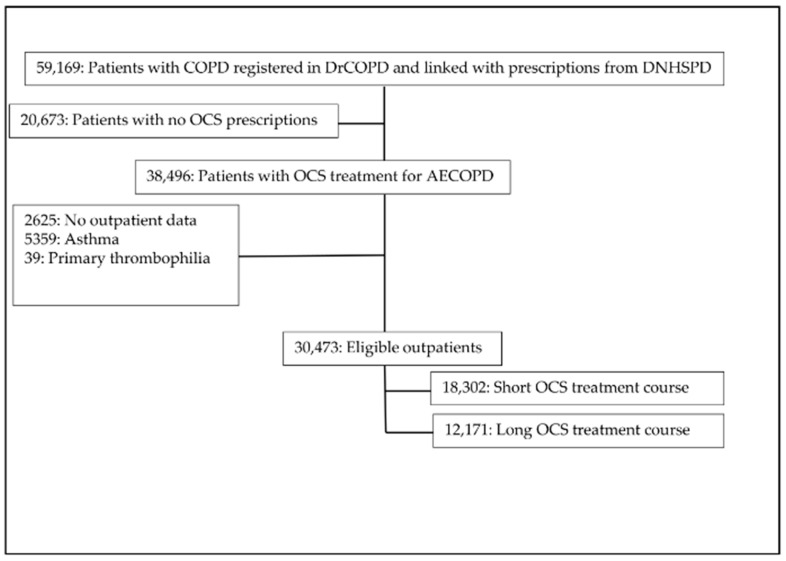
Study flowchart. COPD, chronic obstructive pulmonary disease; AECOPD, acute exacerbations of chronic obstructive pulmonary disease; DNHSPD, Danish National Health Service Prescription Database; DrCOPD, Danish Register of Chronic Obstructive Pulmonary Disease; OCS, oral corticosteroids.

Out of 59,169 COPD outpatients registered in DrCOPD between 1 January 2010 and 28 February 2018, 30,473 COPD patients treated with OCS were identified (Figure 1). These patients were followed for 6 months from the first OCS prescription with a 100% follow-up. The patients were divided as follows: 12,171 (39.9%) received a long OCS treatment course and 18,302 (60.1%) a short OCS treatment course. In total, the median age was 73, with a predominance of women (52.7%) and approximately one-third (37.9%) were active smokers. The groups were similar in age, gender and COPD severity. However, cancer prior to baseline was more prominent in the group with the long course of OCS treatment (*p* < 0.0001); the same was seen with atrial fibrillation (*p* = 0.036), inflammatory bowel disease (*p* < 0.0001) and PE registered prior to baseline (*p* = 0.001). The baseline characteristics are presented in Table 1.

### 3.2. Statistical Analyses

#### 3.2.1. Main Outcome Analysis

**Figure 2:**
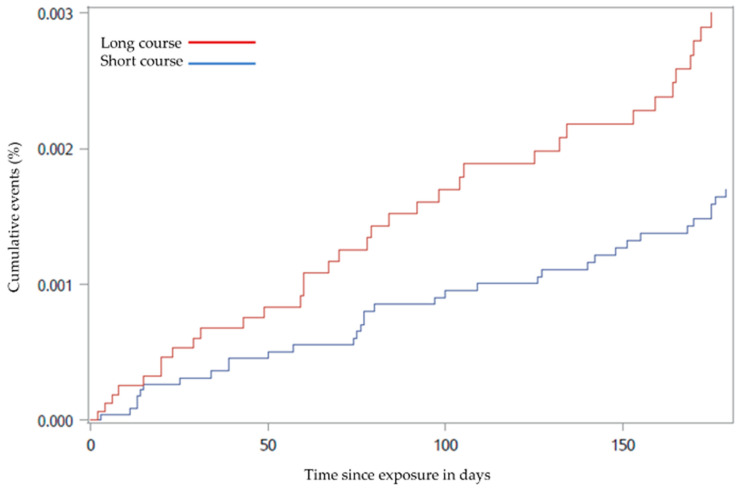
Cumulative incidence curve for venous thromboembolism from baseline until the first event within the 6-month follow-up period among chronic obstructive pulmonary disease outpatients treated with long vs. short oral corticosteroid treatment courses from 2010 to 2018 (*p* = 0.031). The curve is adjusted for death as a competing risk.

**Figure 3:**
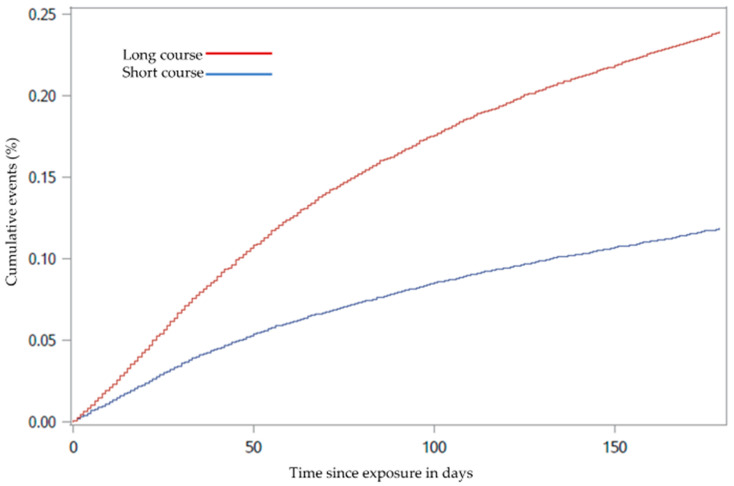
Cumulative incidence curve for all-cause mortality from baseline until the first event within the 6-month follow-up period among chronic obstructive pulmonary disease outpatients treated with long vs. short oral corticosteroid treatment courses from 2010 to 2018 (*p* < 0.0001).

A total of 69 (0.23%) patients had an event of VTE hospitalisation registered within the 6 months they were followed. The incidence rate for VTE hospitalisation was 12 per 1000 days in the long OCS course group and 10.9 per 1000 days in the short OCS course group. The number of events was equally distributed between the two groups: 34 registrations in the short OCS treatment course group and 35 registrations in the long OCS treatment course group. The risk of VTE hospitalisation was similar in the two groups; however, when accounting for death as a competing risk, VTE hospitalisation was higher in the long OCS treatment course group compared to the short OCS treatment course group (HR 1.69, (95% CI 1.05–2.72), *p* = 0.031). In addition, the risk of all-cause mortality was higher in the long OCS treatment course group than the short OCS treatment course group (HR 1.71, (95% CI 1.63–1.79), *p* < 0.0001). Cumulative incidence curves for estimating the probability of VTE hospitalisation and curves estimating the all-cause mortality over 6 months are displayed in Figure 2 and Figure 3.

#### 3.2.2. Sensitivity Analysis

As part of the sensitivity analyses, all COPD patients identified from the DrCOPD registry (*n* = 59,169) were divided into OCS users (*n* = 40,462 (68.4%)) and non-OCS users (*n* = 18,707 (31.6%)). In this sensitivity analysis, all the OCS users were included, asthma and thrombophilia patients as well. The risk of VTE in the two groups was calculated from the first outpatient visit and throughout the 6 months. No VTE hospitalisation events were registered in the non-OCS group. With this sensitivity analysis, we wanted to confirm that the low number of VTE events registered was not explained by the selective population we chose but, rather, reflected a pattern seen in the rest of the COPD population. Secondly, a sensitivity analysis was conducted to investigate the modification effect between OCS use and cancer and the risk of VTE hospitalisation. The presence of cancer significantly modified the risk of VTE hospitalisation caused by OCS (HR 1.4, (95%CI 1.33–1.50), *p* < 0.0001). Thirdly, the time period prior to the guideline switch and after the guideline switch did not change the amount of VTE hospitalisations registered (HR 0.94 (95% CI 0.59–1.75)). Finally, the sensitivity analysis was employed to investigate the modification effect between OCS use and anticoagulation treatment and the risk of VTE hospitalisation. The presence of an anticoagulation treatment did not modify the risk of VTE hospitalisation associated with OCS in a significant way (HR 0.92 (95% CI 0.73–1.81), *p* = 0.477).

## 4. Discussion

In this nationwide observational cohort study including all Danish COPD outpatients, the incidence of VTE hospitalisations in the observation period was lower than expected. When accounting for death as a competing risk, and adjusting for the key confounders, the results showed a 69% increased risk of VTE hospitalisations in COPD patients who received a long OCS treatment course compared to patients who received a short OCS treatment course. Furthermore, a significantly higher risk of all-cause mortality was found in the long OCS treatment course group as compared to the short OCS treatment course group. Supporting the findings illustrated in this study, experimental human and animal studies have shown that OCS treatment increases Factors II, V, VII, IX, X and XII and fibrinogen, thus promoting a hypercoagulability state [19,20]. In asthma patients treated with prednisolone, the von Willebrand factor and Plasminogen activator inhibitor-1 (PAI-1) were increased compared to healthy controls [21]. These investigations could contribute to the clinical practice and increased VTE awareness in COPD patients presenting with dyspnoea. A Danish cohort study presented an increased risk of pneumonia hospitalisation in patients with a long OCS treatment [3]. Pneumonia may have influenced the mortality rate.

Some studies investigating the risk of VTE in COPD patients have been published [12,13,14,22,23,24,25,26], and two meta-analyses have reported a VTE prevalence of up to 25% in hospitalised patients with AECOPD [12,13]. However, previous studies have not investigated OCS as a specific risk factor for VTE but searched specifically for VTE by examining all AECOPD patients admitted to hospital with computed tomographic pulmonary angiography, D-dimers and lower limb ultrasounds [13,23,27]. A recent cross-sectional study demonstrated a 5.9% VTE prevalence in patients with AECOPD within 48 h of admission [28]. In the present study, outpatients were investigated, providing a broader study population, whereas previous studies were more selected, investigating only hospitalised patients. In addition, the number of patients in previous studies was smaller. Further, the difference between our findings and those from the meta-analyses can be explained by the fact that several larger studies that participated in the meta-analyses only included patients with unexplained AECOPD; thus, in reality, a part of these may not have AECOPD but, rather, pulmonary embolism as the cause of admission.

It can be argued that hospitalised COPD patients have more severe COPD, which could explain the higher prevalence reported in the studies mentioned above. Furthermore, hospitalisation itself may also increase the VTE risk due to immobility.

Data from a multicentre clinical registry (RIETE) included patients with confirmed symptomatic VTE (*n* = 28,920). Out of the VTE patients, 2984 (10.3%) had COPD as a comorbidity [22]. COPD was associated with a higher risk of PE than in non-COPD patients (odds ratio (OR) 1.64, (95% CI 1.49–1.80)). The overall mortality was significantly higher in COPD patients (2.6%) than in non-COPD patients (1.7%). PE was the cause of death in the majority of COPD patients (66.7%). The results from the RIETE thus suggested that the chronicity and variability of symptoms, and the frequent exacerbations in COPD patients, might have led to the underdiagnosis of PE in this patient group. The diagnosis of PE is a clinical challenge and may be particularly challenging in COPD patients, because the symptoms and signs caused by PE and AECOPD are often similar.

However, this association has been studied in patients with other conditions, including upper and lower respiratory tract diseases, allergic reactions, bronchitis and spinal conditions, as well as post-surgery [9,10,11,15,29]. A case–control study using a Dutch population-based pharmacy registry reported an association between OCS use and the risk of hospitalisation due to PE. The cases (patients with a first hospital admission for PE) were compared to the controls (sex- and age-matched subjects without a history of PE). The association between OCS and PE increased with the dose of OCS, thus establishing a solid dose–response relationship [29]. In a neurosurgical population, prolonged OCS use (>30 days) was associated with an increased risk of developing postoperative VTE (2.1% in the OCS group and 0.7% in the control group that did not receive OCS) [30]. Similar results were found in other postoperative patient groups [10,11]. The present study used the Danish guideline switch from 2014 to make the groups comparable, since 10- and 5-day OCS treatments were the first-line treatments in their respective time periods. However the aforementioned studies compared OCS users with non-OCS users, it can be argued that the patient group receiving OCS were more ill compared to the controls.

The high prevalence (25%) of PE in COPD patients illustrated in the previously mentioned meta-analysis was not observed in the present study. The present study was observational; therefore, only patients who were diagnosed and registered with a VTE diagnosis could be detected. This suggests a possible underdiagnoses of PE in patients registered in DrCOPD. However, based on our data, we are unfortunately unable to comment on whether it was a mild, moderate or severe VTE. Similarly, other studies have brought attention to the fact that, because of an overlap in symptoms, PE might be overlooked in COPD patients, because AECOPD is a more likely diagnosis [31]. Autopsy studies have shown that the clinical diagnosis of PE was suspected less frequently in patients with concomitant COPD [14,32]. The present data underlined the need to systematically examine patients with COPD for PE whenever this condition is suspected, especially in patients where the reason for AECOPD is unclear and patients who have recently started OCS treatment. Furthermore, this study stresses the need for precise data registration whenever PE is diagnosed in COPD patients.

### Strengths and Limitations

The large sample size and the unselected and well-characterised population of patients with a COPD diagnosis verified at every outpatient visit by a specialist in pulmonary medicine represented the important strengths of the present study. Furthermore, in Denmark, getting OCS requires a prescription, and all prescriptions are registered in the national prescription database, which thus assured a high degree of data completeness. Additionally, hospital admissions and deaths are registered; this secured the totality of data registration and follow-up. Nevertheless, our study had important limitations. Firstly, the small number of VTE events registered (*n* = 69) increased the risk of chance findings; in this case, a Type I error [31]. Secondly, despite the use of a guideline shift to minimise the diversity in the two groups, the possibility of confounding by indication was still present, i.e., more ill patients possibly received longer courses of OCS.

In conclusion, we found that a 10-day course of OCS was associated with a substantially increased risk of VTE hospitalisation in COPD patients when compared to 5-day courses. This supports the current strategy to minimise the use of systemic corticosteroids. However, the limited number of registered events leaves open the possibility for chance findings or misclassification bias.

## Figures and Tables

**Table 1:** Baseline characteristics of outpatients with chronic obstructive pulmonary disease who were treated with a long vs. short oral corticosteroid (OCS) course during the period of January 2010–February 2018.

N (Number of Participants)	Total (*n* = 30,473)	Short OCS Treatment Course (*n* = 18,302)	Long OCS Treatment Course (*n* = 12,171)
Age, years, median (IQR)	73 (66–80)	73 (66–80)	73 (66–79)
Age			
≤62 years	6275 (20.6%)	3781 (20.7%)	2494 (20.5%)
63–70 years	8081 (26.5%)	4713 (25.8%)	3368 (27.7%)
71–77 years	7977 (26.2%)	4745 (25.9%)	3232 (26.6%)
≥78 years	8140 (26.7%)	5063 (27.7%)	3077 (25.3%)
Sex			
Female	16,074 (52.7%)	9830 (53.7%)	6244 (51.3%)
Male	14,399 (47.3%)	8472 (46.3%)	5927 (48.7)
Smoking			
Active	11,553 (37.9%)	7155 (39.1%)	4398 (36.1%)
Former/never smoker	18,219 (59.8%)	10,702 (58.5%)	7517 (61.8%)
Missing value	701 (2.3%)	445 (2.4%)	256 (2.1%)
BMI, kg/m^2^, median (IQR)	25 (21–29)	25 (21–29)	24 (21–28)
BMI			
≤18.4 kg/m^2^	3683 (12.1%)	2098 (11.5%)	1585 (13.0%)
18.5–24.9 kg/m^2^	12,158 (39.9%)	7142 (39.0%)	5016 (41.2%)
25–30 kg/m^2^	8291 (27.2%)	5093 (27.8%)	3198 (26.3%)
>30 kg/m^2^	6341 (20.8%)	3969 (21.7%)	2372 (19.5%)
FEV_1_			
≥80%	1211 (3.9%)	760 (4.2%)	451 (3.7%)
<80–50%	9989 (32.8%)	6405 (35.0%)	3584 (29.4%)
<50–30%	10,906 (35.8%)	6561 (35.8%)	4345 (35.7%)
<30%	8367 (27.5%)	4576 (25.0%)	3791 (31.1%)
Exacerbations 12 months prior to baseline	0	10,947 (59.8%)	6080 (50.0%)
	1	2798 (15.3%)	2186 (18.0%)
	≥2	4557 (24.9%)	3905 (32.1%)
Total dose of oral corticosteroids		250 (12.5–250)	500 (500–7500)
Comorbidities *			
Pulmonary embolism	1284 (4.2%)	716 (3.9%)	568 (4.7%)
Deep vein thrombosis	962 (3.2%)	566 (3.1%)	396 (3.3%)
Inflammatory Bowel disease	581 (1.9%)	303 (1.7%)	278 (2.3%)
Malignancy	5633 (18.5%)	2829 (15.5%)	2804 (23.0%)
Diabetes mellitus	3854 (12.6%)	2322 (12.7%)	1532 (12.6%)
Hypertension	10,791 (35.4%)	6517 (35.6%)	4274 (35.1%)
Myocardial infarction	2533 (8.3%)	1506 (8.2%)	1027 (8.4%)
Peripheral vascular disease	3837 (12.5%)	2354 (12.9%)	1483 (12.2%)
Cerebrovascular disease	3065 (10.1%)	1837 (10.0%)	1228 (10.1%)
Renal failure	1690 (5.5%)	996 (5.4%)	694 (5.7%)
Heart failure	5094 (16.7%)	3008 (16.4%)	2086 (17.1%)
Depression	1072 (3.5%)	672 (3.7%)	400 (3.3%)
Atrial fibrillation	5773 (18.9%)	3397 (18.6%)	2376 (19.5%)

* All comorbidities registered within 10 years prior to the first OCS prescription.

## Data Availability

We believe that knowledge sharing increases the quality and quantity of scientific results. The sharing of relevant data will be discussed within the study group upon reasonable request.
